# Supplementary material for: The Impact of Protein Glycosylation on the Identification of Patients with Pediatric Appendicitis
Source: Int J Mol Sci. 2024 Jun 11;25(12):6432. doi: 10.3390/ijms25126432 (PMC11204372; doi:10.3390/ijms25126432)
Supplement: Supplementary file 1 [file ijms-25-06432-s001.zip › ijms-3018521-supplementary.pdf]

Supplementary

The Impact of Protein Glycosylation in The Identification of Patients with Pediatric Appendicitis

Dalma Dojcsák [1], Flóra Farkas [2], Tamás Farkas [2], János Papp [2], Attila Garami[3], Béla Viskolcz [1,4] and Csaba Váradi [4], *

1 Advanced Materials and Intelligent Technologies Higher Education and Industrial Cooperation Centre, University of Miskolc, Hungary 3515;
2 Borsod-Abaúj-Zemplén County Center Hospital and University Teaching Hospital, Miskolc, Hungary 3526
3 Institute of Energy, Ceramic and Polymer Technology, University of Miskolc, Hungary 3515
4 Institute of Chemistry, Faculty of Materials Science and Engineering, University of Miskolc, Hungary 3515
* Correspondence: csaba.varadi@uni-miskolc.hu; Tel.: +30-894-7730 (Cs.V.)

Supplementary Table S1. Summary data of the identified glycan structures with the retention time, theoretical m/z, average area% + standard deviation, statistic value and significance level of Shapiro-Wilk test and D'Agostino's K2 test, significance level of Kruskal-Wallis test. The GlycoWorkBench software was used for the identification of N-glycans based on the measured and theoretical m/z ratio and structural parameters of the glycans.

Retention time [min]	*Glycan structure name [Theoretical m/z]$^{2+}$*	*#Peak number*	*Average area% of Appendicitis*	*Std. dev.*	*Average area% of Normal control*	*Std. dev.*	*Average area% of Abdominal pain control*	*Std. dev.*	*Shapiro-Wilk test (*p<0.05; **p<0.01)*	*D'Agostino's K^2 test (*p<0.05; **p<0.01)*	*Significance level Kruskal-Wallis test_Control vs Appendicitis (**p<0.01; *** p< 0.001)*
15.06	*FA2* [841.94]$^{2+}$	*#1*	2.43	1.94	1.53	1.07	4.60	3.80	**1.47E-04	**5.49E-05	0.04
16.13	*M5* [727.89]$^{2+}$	*#2*	2.09	0.37	1.06	0.17	2.20	1.83	**6.50E-07	**1.02E-10	0.01
16.35	*FA2B* [943.48]$^{2+}$	*#3*	2.22	0.28	1.16	0.25	3.00	2.15	*1.19E-02	**2.33E-02	**0.001
17.58	*FA2G1* [922.97]$^{2+}$	*#4*	2.29	1.32	1.34	0.86	3.70	2.51	*1.71E-02	**1.53E-03	**0.006
17.95	*FA2G1* [922.97]$^{2+}$	*#5*	2.60	0.60	1.76	0.43	6.40	4.31	**1.95E-03	**1.10E-03	**0.004
18.60	*FA2BG1* [1024.51]$^{2+}$	*#6*	3.28	0.26	2.53	0.19	11.60	7.01	2.22E-02	1.10E-01	**0.005
18.64	*FA2BG1* [1024.51]$^{2+}$	*#7*	2.26	0.13	1.30	0.07	3.90	3.62	**3.06E-06	**2.21E-10	***<0.001
19.02	*M6* [808.91]$^{2+}$	*#8*	2.26	0.33	1.41	0.19	4.70	3.85	**4.55E-06	**3.15E-07	0.27
19-2	*A2G2* [930.97]$^{2+}$	*#9*	2.60	0.28	1.74	0.23	6.10	2.85	1.52E-01	**1.64E-02	***<0.001
19.95	*A2BG2* [1032.51]$^{2+}$	*#10*	2.45	0.05	1.54	0.05	5.13	3.50	7.26E-01	4.06E-01	0.92
20.15	*A2G1S1* [995.49]$^{2+}$	*#11*	0.55	0.12	0.58	0.08	2.77	1.16	*1.25E-02	**2.62E-03	0.14
20.32	*FA2G2* [1003.92]$^{2+}$	*#12*	3.21	0.93	2.71	0.82	13.60	8.33	6.02E-01	2.02E-01	***<0.001
20.86	*A2G2S1* [1076.51]$^{2+}$	*#13*	3.08	0.21	2.46	0.28	11.80	7.31	6.60E-02	1.55E-01	0.12
20.95	*FA2BG2* [1105.53]$^{2+}$	*#14*	3.59	0.23	3.09	0.25	13.50	7.80	1.32E-01	2.62E-01	0.26
21.11	*M4G1S1* [974.97]$^{2+}$	*#15*	2.44	0.17	1.56	0.12	5.19	3.62	7.77E-01	8.60E-01	***<0.001
21.35	*M7* [889.94]$^{2+}$	*#16*	3.19	0.05	2.62	0.07	12.48	7.31	**1.34E-03	**7.75E-04	**0.001

21.83	*M7* [889.94] $^{2+}$	*#17*	2.48	0.03	1.75	0.04	8.41	5.08	2.13E-01	3.56E-01	***<0.001
22.30	*A2G2S1* [1076.51] $^{2+}$	*#18*	3.05	2.59	2.36	0.99	8.80	5.51	**5.05E-10	**3.79E-19	0.07
22.44	*A2G2S1* [1076.51] $^{2+}$	*#19*	2.65	0.06	1.97	0.09	9.20	5.58	**1.44E-05	**3.80E-26	***<0.001
23.25	*A2FG2S1* [1149.54] $^{2+}$	*#20*	2.53	0.93	1.69	0.67	8.40	5.12	3.60E-01	7.89E-01	***<0.001
23.66	*FA2G2S2* [1295.09] $^{2+}$	*#21*	2.28	0.16	1.38	0.13	4.20	3.17	2.17E-01	1.58E-01	**0.001
23.98	*A2G2S2* [1222.06] $^{2+}$	*#22*	2.58	0.68	1.74	0.75	7.00	4.76	6.97E-01	5.23E-01	0.34
24.22	*M8* [970.88] $^{2+}$	*#23*	2.03	0.06	0.97	0.08	1.60	1.60	*3.81E-03	6.85E-02	***<0.001
24.76	*FA2G2S2* [1295.09] $^{2+}$	*#24*	2.94	0.09	2.24	0.07	10.00	6.65	4.66E-01	7.17E-01	***<0.001
25.12	*A2G2S2* [1222.06] $^{2+}$	*#25*	2.43	3.90	1.55	1.77	4.40	3.36	*1.56E-02	6.42E-02	***<0.001
25.75	*FA2G2S* [1295.09] $^{2+}$	*#26*	2.57	0.29	1.77	0.23	5.30	3.14	**7.54E-04	**7.70E-06	***<0.001
25.97	*FA2G2S2* [1295.09] $^{2+}$	*#27*	2.69	0.64	2.00	0.95	7.90	5.29	**6.10E-07	**2.55E-12	0.84
26.34	*FA2BG2S2* [1396.63] $^{2+}$	*#28*	2.67	0.30	1.81	0.23	6.50	3.95	*2.24E-02	1.04E-01	**0.002
26.61	*A2BG3S2* [1404.63] $^{2+}$	*#29*	3.08	0.34	2.40	0.33	10.30	6.33	1.12E-01	*2.42E-02	***<0.001
26.92	*A2BG3S2* [1404.63] $^{2+}$	*#30*	3.70	0.08	3.14	0.03	13.90	5.65	**1.73E-04	**8.92E-05	0.87
27.75	*A2BG3S2* [1404.63] $^{2+}$	*#31*	3.49	0.23	2.98	0.34	14.20	8.37	**2.52E-03	*9.15E-02	***<0.001
27.83	*FA3G3S2* [1477.65] $^{2+}$	*#32*	3.05	0.15	2.59	0.22	10.80	5.55	**1.14E-02	**3.71E-03	**0.002
28.05	*A3G3S3* [1550.17] $^{2+}$	*#33*	2.87	0.29	2.03	0.19	7.20	5.41	**2.62E-04	**1.32E-06	0.03
28.05	*A3G3S3* [1550.18] $^{2+}$	*#34*	2.59	0.19	1.84	0.10	7.40	5.00	**9.41E-06	**9.53E-05	***<0.001
28.40	*A3G3S3* [1550.18] $^{2+}$	*#35*	2.38	0.19	1.41	0.08	3.20	2.97	**1.67E-05	**3.84E-05	**0.001
28.97	*A3G3S3* [1550.18] $^{2+}$	*#36*	3.03	1.20	2.41	1.07	9.80	7.30	2.25E-01	6.13E-01	0.86
29.24	*A4G4S2* [1587.19] $^{2+}$	*#37*	2.46	0.31	1.56	0.17	3.70	3.74	*1.43E-02	**1.66E-03	0.05
29.52	*FA3G3S3* [1623.20] $^{2+}$	*#38*	3.14	0.11	2.46	0.11	10.00	5.19	2.22E-01	7.56E-01	0.10
30.01	*A3G3S3* [1550.18] $^{2+}$	*#39*	3.17	0.43	2.69	0.39	12.70	7.67	2.31E-01	4.38E-01	0.21
30.10	*FA3G3S3* [1623.20] $^{2+}$	*#40*	2.52	1.36	1.75	0.67	6.20	3.58	**4.28E-07	**1.16E-10	0.04
30.34	*A4G4S3* [1732.74] $^{2+}$	*#41*	2.82	0.30	2.10	0.21	8.54	5.27	9.80E-01	9.88E-01	0.28
30.53	*FA3FG3S3* [1696.24] $^{2+}$	*#42*	0.67	0.11	0.76	0.08	3.50	1.35	**1.76E-07	**7.77E-11	0.18
31.20	*A4G4S3* [1732.74] $^{2+}$	*#43*	6.99	0.23	7.45	0.12	5.30	66.17	**3.09E-03	**2.83E-04	0.48
31.50	*A4G4S4* [1878.29] $^{2+}$	*#44*	3.79	0.28	3.27	0.17	16.40	7.95	*1.52E-02	*2.64E-02	0.46
32.20	*A4G4S4* [1878.29] $^{2+}$	*#45*	12.35	0.33	16.05	0.16	8.40	164.55	**4.33E-05	**1.27E-04	0.15
32.40	*A4G4S4* [1878.29] $^{2+}$	*#46*	3.70	0.19	3.30	0.09	8.50	9.24	**9.90E-07	**3.94E-10	0.1
33.01	*FA4G4S4* [1951.32] $^{2+}$	*#47*	5.13	0.35	5.32	0.13	12.00	29.31	**1.41E-09	**1.61E-12	0.34

33.67	*FA4G4S4* [1951.32] $^{2+}$	*#48*	4.76	0.09	4.79	0.03	14.30	13.38	**1.37E-08	**1.23E-12	0.54

Supplementary Table S2. Summary table of the identified 4 significantly changed glycan structures based on the Kruskal-Wallis test and ROC analysis. In concluison, area% values can be defined as a threshold value, which classify samples into the diseased or the control groups based on a specificity and a sensitivity percentage values.

Structure name	Average area% ± std	AUC	Sensitivity/ Specificity	Kruskal-Wallis test	Conclusion
A2G2S2#19	0.42 ± 0.04	0.98	94% / 100%	p<0.001	If the area % is higher than 0.44, the sample is described the control group with 94% sensitivity and 100% specificity
A2BG3S2#31	1.07 ± 0.22	0.97	91% / 100%	p<0.001	If the area % is higher than 0.84, the sample is described the control group with 91% sensitivity and 100% specificity
A3G3S3#34	0.40 ± 0.06	0.98	94% / 100%	p<0.001	If the area % is higher than 0.25, the sample is described the patient group with 94% sensitivity and 100% specificity
A2G2S2#25	32.35 ± 2.51	0.89	79% / 100%	p<0.001	If the area % is higher than 25.2, the sample is described the patient group with 79% sensitivity and 100% specificity

Supplementary Table S3. The principle laboratory data for the samples collected in collaboration with Department of Pediatric Surgery in Borsod-Abaúj-Zemplén County Center Hospital Miskolc, Hungary, are summerized in the table below.

Group	Sample number	CRP [mg/L] (Av. ± std. dev.)	WBC [g/L] (Av. ± std. dev.)	*ANC [g/L]* (Av. ± std. dev.)	Gender	Av. Age
Abdominal pain control	9	1,24±0,83	8,24±2,90	5,13±2,77	5 female 4 male	13
Normal control	29	1,48±1,26	11,77±3,7	8,54±3,5	7 female 22 male	9
Appendicitis	40	70,72±76,1	15,63±5,51	12,67±5,4	9 female 31 male	11

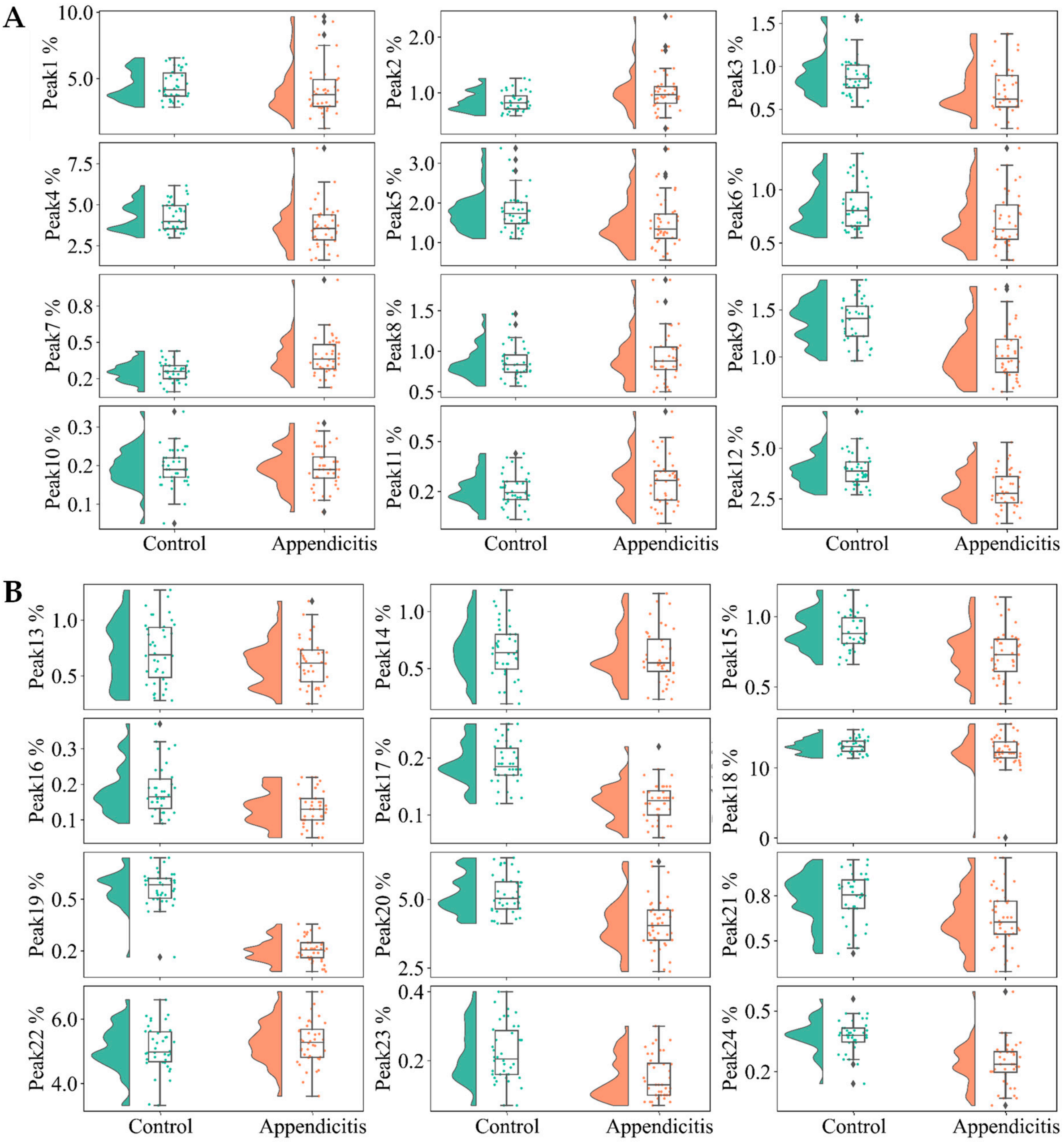
A
Peak1 %
Peak2 %
Peak3 %
Peak4 %
Peak5 %
Peak6 %
Peak7 %
Peak8 %
Peak9 %
Peak10 %
Peak11 %
Peak12 %
Control
Appendicitis
B
Peak13 %
Peak14 %
Peak15 %
Peak16 %
Peak17 %
Peak18 %
Peak19 %
Peak20 %
Peak21 %
Peak22 %
Peak23 %
Peak24 %
Control
Appendicitis

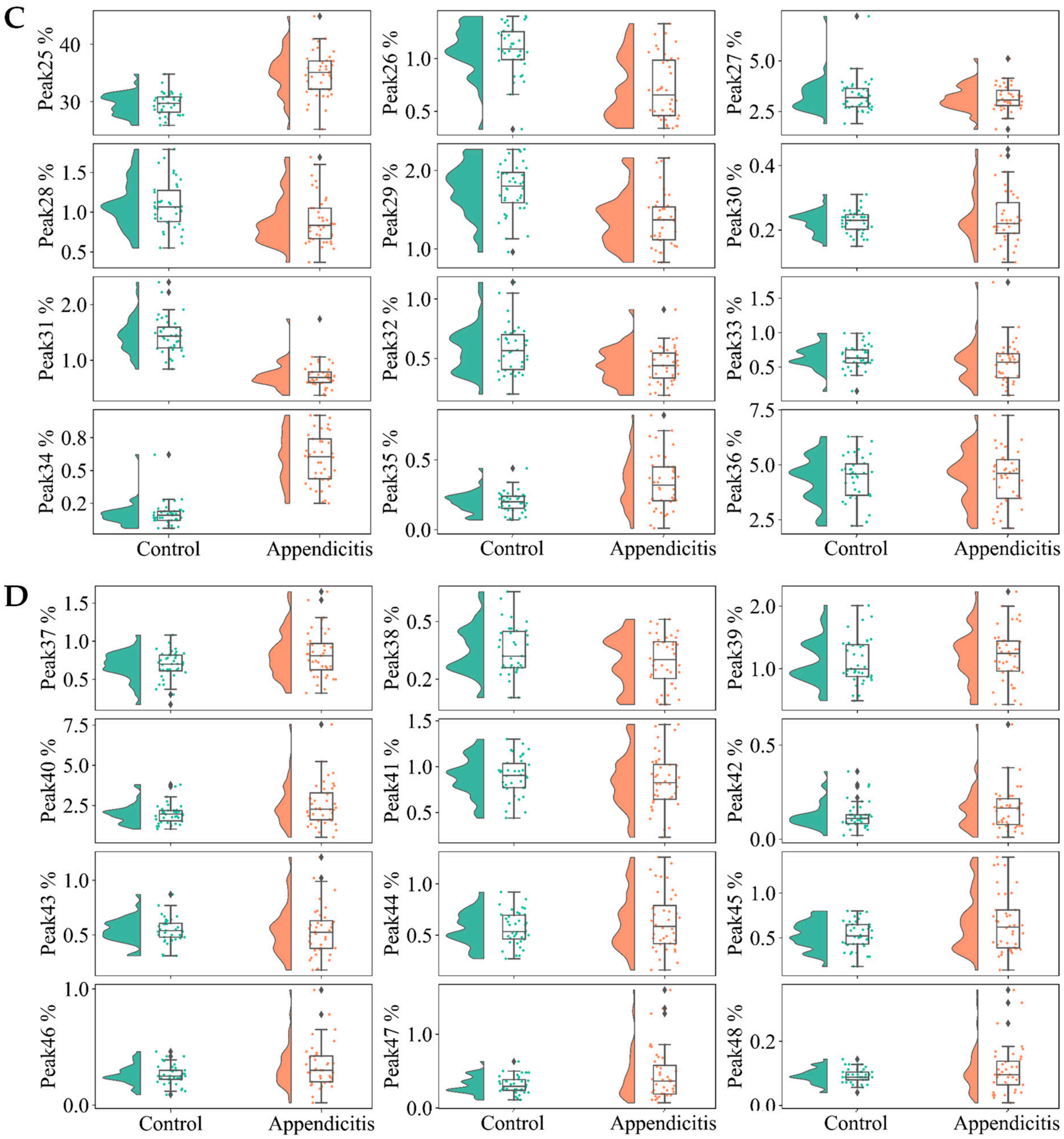

Supplementary Figure S1. The boxplot chart was created to display a summary of a set of area% data values with minimum, first quartile, median, third quartile and maximum properties. Distribution curves showed that in most cases our data cannot be described by normal distribution. Identified structures are labelled by Peak1-48. On x-axis the area% of the structures labelled. A: distribution curve and boxplot diagtam of glycan structures Peak1-12; B: distribution curve and boxplot diagtam of glycan structures Peak13-24; C: distribution curve and boxplot diagtam of glycan structures Peak25-36; D: distribution curve and boxplot diagtam of glycan structures Peak37-48.

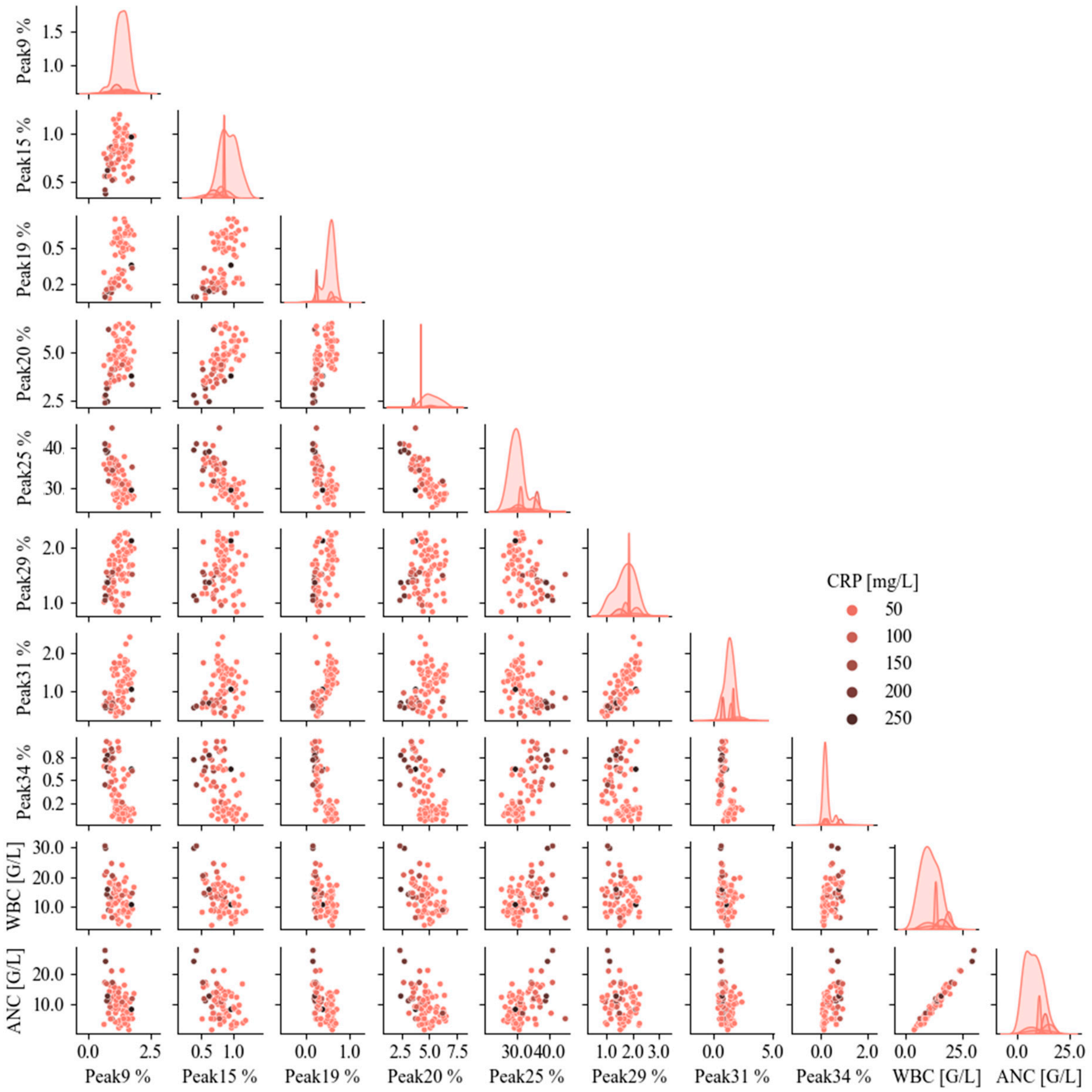

Supplementary Figure S2. Pair plot diagrams of significantly different glycan structures depending on CRP value. The pairplot diagrams presence the distribution of samples across various CRP value intervals according the glycan area% values. In case of Peak19 (A2G2S2#19) the samples with lower CRP value separate into a group, which pattern is similar to the pairplot of disease score (Figure 3.). Higher area% level of peak19 correlate with lower CRP value, additionally the A2G2S1#19 area% shows higher level in case of control group.

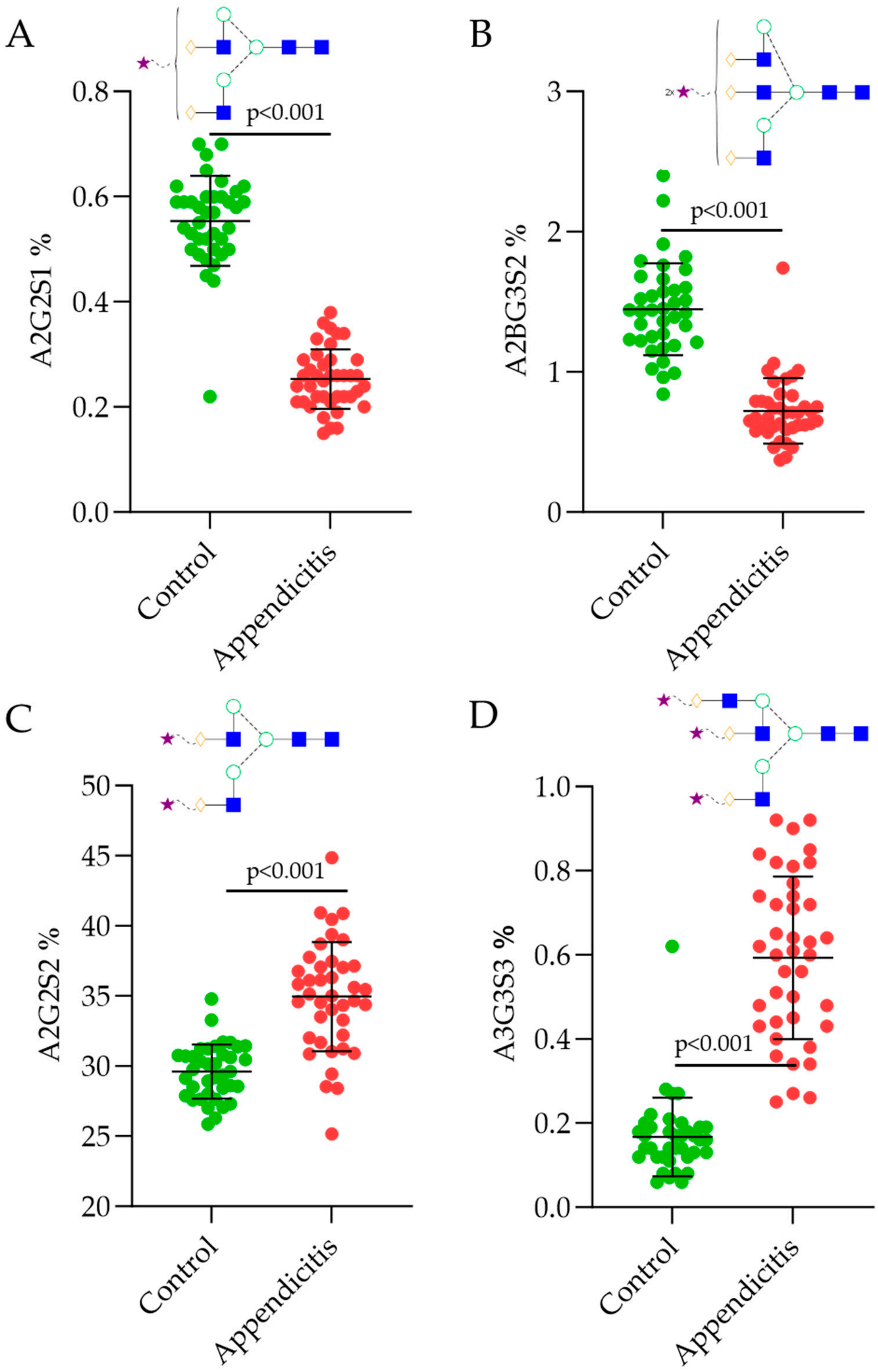

Supplementary Figure S3. Based on the ROC analysis and Kruskal-Wallis test, 4 glycan structures were significantly altered in appendicitis group. A: A2G2S1#19 level is decreased; B: A2BG3S2#31 level is decreased; C: A3G3S3#34 level is increased; D: A2G2S2#25 level is increased

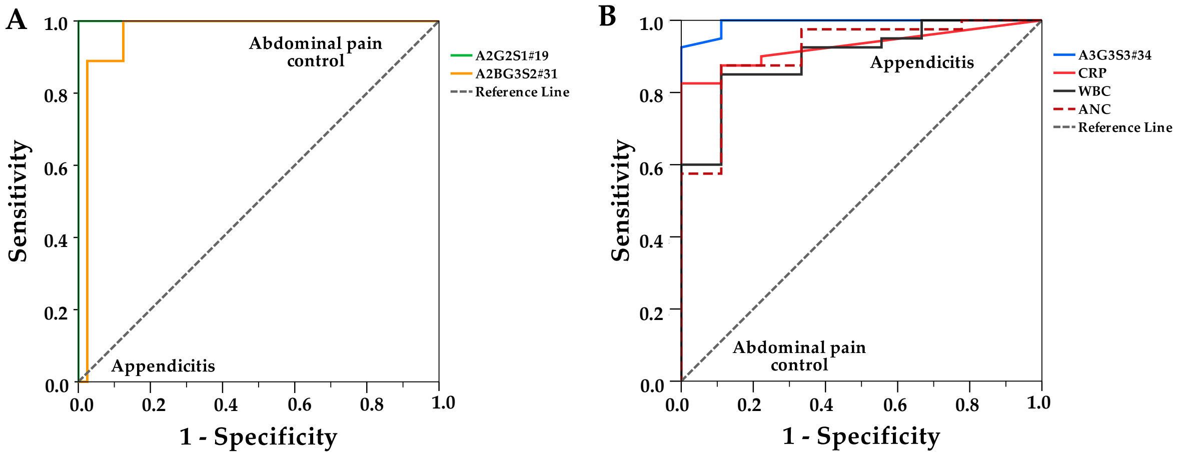

Test variables	AUC	Std. Error	Asymptotic 99% Confidence Interval	
			Sensitivity	Specificity
A2G2S1#19	1.00	0.01	100%	100%
A2BG3S2#31	0.96	0.03	91%	100%
A3G3S3#34	0.99	0.01	98%	100%
CRP	0.93	0.04	86%	100%
ANC	0.91	0.05	82%	100%
WBC	0.90	0.05	80%	100%

Supplementary Figure S4. ROC curves for the glycan peaks which significantly changed with abdominal pain control (A) or appendicitis groups (B). Null hypothesis: true area=0.5. Confidence level was 99%. Significance levels were below 0.001 in each cases.

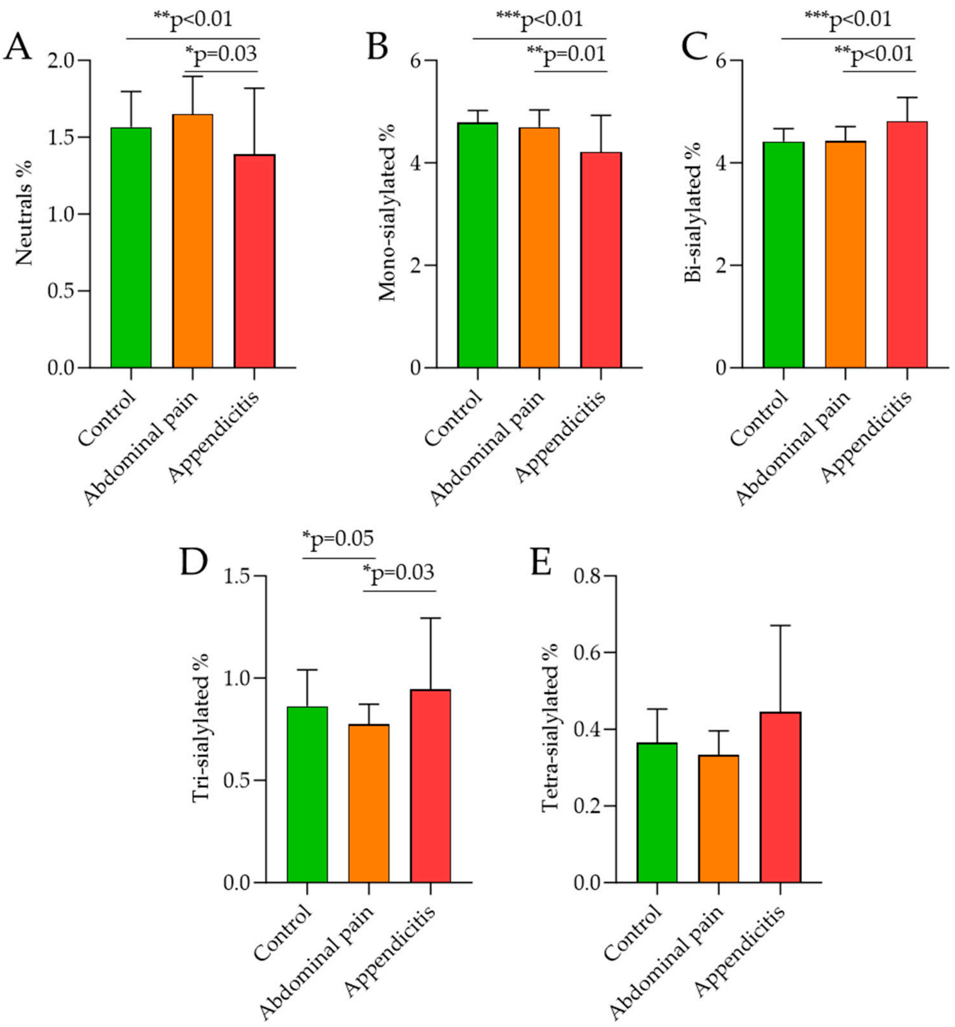

Supplementary Figure S5. Lower level of neutral glycans (A) and mono- sialylated (B) glycans while higher sialylation on bi- (C), tri- (D) and tetra-antennary glycans. The x-axis repsresents the area% values of various glycan structures.

A,

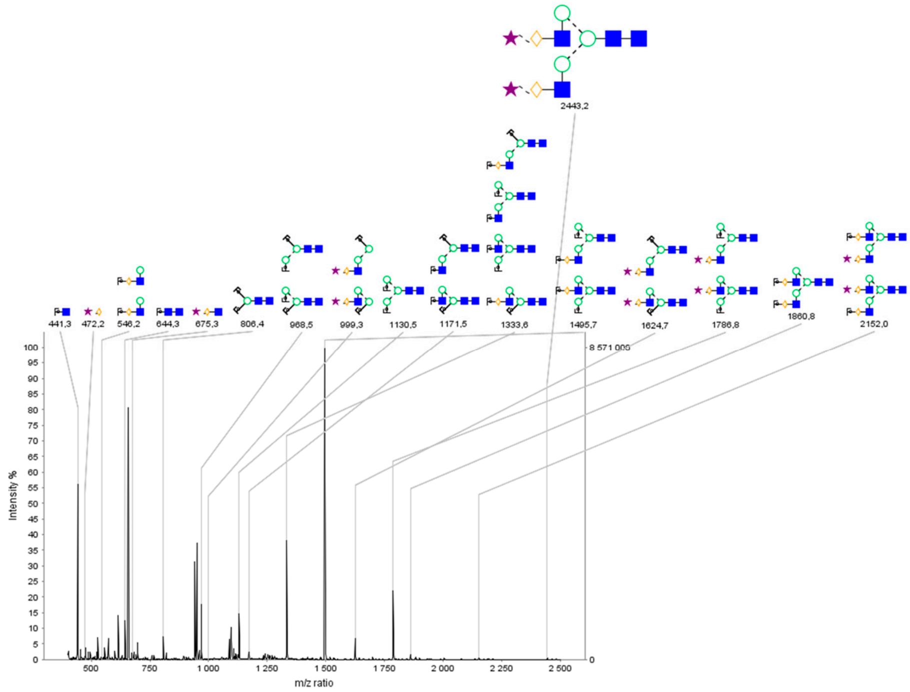

B,

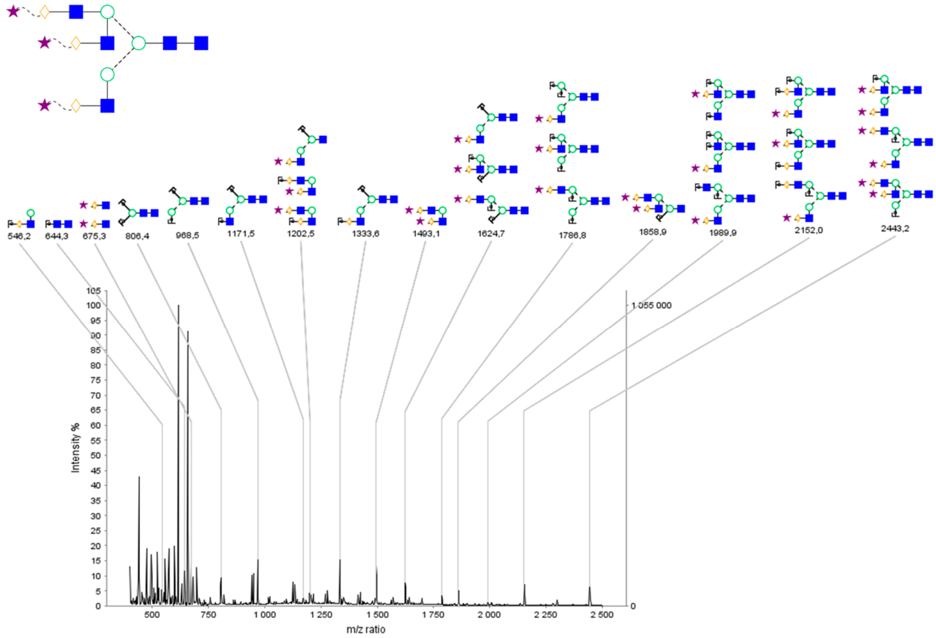

Supplementary Figure S6. The structural identification was perfomed by MS/MS analysis and subsequent annotaion. A: Fragmentation of parent ion: A2G2S2 [2443.2]$^{+}$; B: Fragmentation of parent ion: A3G3S3 [3099.3]$^{+}$.
